# Supplementary material for: Hour-1 bundle adherence was associated with reduction of in-hospital mortality among patients with sepsis in Japan
Source: PLoS One. 2022 Feb 14;17(2):e0263936. doi: 10.1371/journal.pone.0263936 (PMC8843226; doi:10.1371/journal.pone.0263936)
Supplement: S2 Fig — Univariate and multivariate-adjusted ORs with 95% CIs for mortality risk are represented as forest plots. Covariate adjustment or propensity score adjustment was used in the regression analyses as appropriate. OR odds ratio, CI confidence interval, PS propensity score. (DOCX) [file pone.0263936.s002.docx]

**
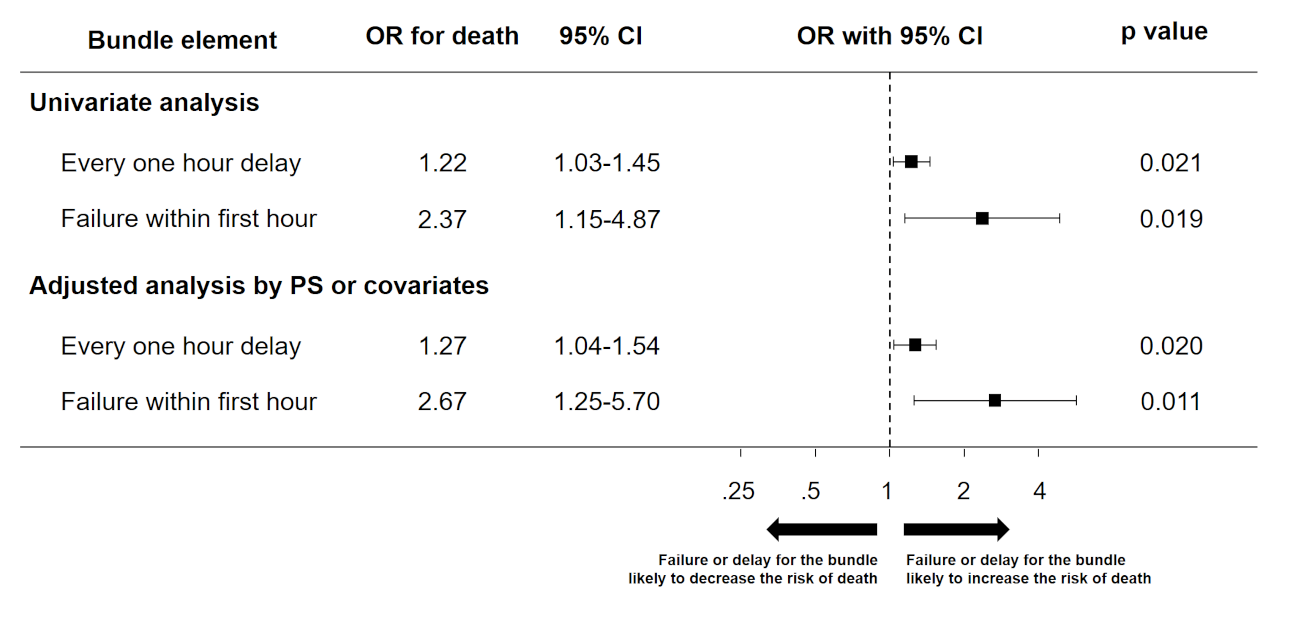
**

**S2 Fig. Association between the increase in mortality and failure or delay in achieving three relatively low-compliance bundle components: blood culture, antibiotics, and vasopressors.**
