# Supplementary material for: Hour-1 bundle adherence was associated with reduction of in-hospital mortality among patients with sepsis in Japan
Source: PLoS One. 2022 Feb 14;17(2):e0263936. doi: 10.1371/journal.pone.0263936 (PMC8843226; doi:10.1371/journal.pone.0263936)
Supplement: S1 Table — SOFA Sequential Organ Failure Assessment, ICU intensive care unit. (DOCX) [file pone.0263936.s003.docx]

| **S1 Table. The 11 variables used to calculate propensity scores for adherence to the hour-1 bundle in the logistic regression models.** | |
| --- | --- |
| Patient characteristics | Age, sex |
| Illness severity | SOFA neurological subscore, SOFA cardiovascular subscore, SOFA coagulation subscore, SOFA hepatic subscore, SOFA renal subscore, SOFA respiratory subscore, mechanical ventilation use |
| Source of ICU admission | Emergency department/Onset in another ward/Onset in ICU |
| Pre-existing condition | Charlson comorbidity index |
| *SOFA* Sequential Organ Failure Assessment, *ICU* intensive care unit | |
