# Supplementary material for: Hour-1 bundle adherence was associated with reduction of in-hospital mortality among patients with sepsis in Japan
Source: PLoS One. 2022 Feb 14;17(2):e0263936. doi: 10.1371/journal.pone.0263936 (PMC8843226; doi:10.1371/journal.pone.0263936)
Supplement: S2 Table — Missing data: Obtain blood cultures = 4; Administration of crystalloid = 1; Apply vasopressors = 2; completion of all elements = 1. (DOCX) [file pone.0263936.s004.docx]

| **S2 Table. Time to completion of each component of the hour-1 bundle.** | | | | | | | | |
| --- | --- | --- | --- | --- | --- | --- | --- | --- |
| Components of hour-1 bundle | Not applicable | < 1 hour | 1-2 hours | 2-3 hours | 3-4 hours | 4-5 hours | 5-6 hours | > 6 hours |
| Measure lactate level | - | 176 (98.9%) | 2 (1.1%) | 0 (0%) | 0 (0%) | 0 (0%) | 0 (0%) | 0 (0%) |
| Obtain blood cultures | - | 150 (86.2%) | 17 (9.8%) | 3 (1.7%) | 1 (0.6%) | 3 (1.7%) | 0 (0%) | 0 (0%) |
| Broad-spectrum antibiotics | - | 91 (51.1%) | 40 (22.5%) | 20 (11.2%) | 5 (2.8%) | 6 (3.4%) | 4 (2.3%) | 12 (6.7%) |
| Administration of crystalloid | 59 | 112 (94.9%) | 3 (2.5%) | 2 (1.7%) | 0 (0%) | 0 (0%) | 0 (0%) | 1 (0.9%) |
| Apply vasopressors | 79 | 67 (69.1%) | 15 (15.5%) | 5 (5.2%) | 3 (3.1%) | 3 (3.1%) | 1 (1%) | 3 (3.1%) |
| Completion of all components | - | 89 (50.3%) | 35 (19.8%) | 21 (11.9%) | 7 (4%) | 7 (4%) | 5 (2.8%) | 13 (7.3%) |
| Missing data: Obtain blood cultures = 4; Administration of crystalloid = 1; Apply vasopressors = 2; completion of all elements = 1 | | | | | | | | |
