# Supplementary material for: Hour-1 bundle adherence was associated with reduction of in-hospital mortality among patients with sepsis in Japan
Source: PLoS One. 2022 Feb 14;17(2):e0263936. doi: 10.1371/journal.pone.0263936 (PMC8843226; doi:10.1371/journal.pone.0263936)
Supplement: S3 Table — (DOCX) [file pone.0263936.s005.docx]

| **S3 Table. Time to completion of each component of the hour-1 bundle in the non-bundle-adherent group.** | | | | | | | | |
| --- | --- | --- | --- | --- | --- | --- | --- | --- |
| Components of hour-1 bundle | Not applicable | < 1 hour | 1-2 hours | 2-3 hours | 3-4 hours | 4-5 hours | 5-6 hours | > 6 hours |
| Measure lactate level | - | 88 (98.9%) | 1 (1.1%) | 0 (0%) | 0 (0%) | 0 (0%) | 0 (0%) | 0 (0%) |
| Obtain blood cultures | - | 62 (72.1%) | 17 (19.8%) | 3 (3.5%) | 1 (1.2%) | 3 (3.5%) | 0 (0%) | 0 (0%) |
| Broad-spectrum antibiotics | - | 5 (5.6%) | 39 (43.8%) | 20 (22.5%) | 4 (4.5%) | 6 (6.7%) | 4 (4.5%) | 11 (12.4%) |
| Administration of crystalloid | 31 | 52 (89.7%) | 3 (5.2%) | 2 (3.5%) | 0 (0%) | 0 (0%) | 0 (0%) | 1 (1.7%) |
| Apply vasopressors | 37 | 22 (42.3%) | 15 (28.9%) | 5 (9.6%) | 3 (5.8%) | 3 (5.8%) | 1 (1.9%) | 3 (5.8%) |
| Completion of all components | - | 0 (0%) | 35 (39.8%) | 21 (23.9%) | 7 (8%) | 7 (8%) | 5 (5.7%) | 13 (14.8%) |
